# Effects of Jet Path on Electrospun Polystyrene Fibers

**DOI:** 10.3390/polym10080842

**Published:** 2018-07-30

**Authors:** Yuansheng Zheng, Na Meng, Binjie Xin

**Affiliations:** School of Fashion Engineering, Shanghai University of Engineering Science, Shanghai 201620, China; 15257305824@163.com

**Keywords:** electrospinning, jet path, solution concentration, fiber morphology

## Abstract

In this study we investigated the effects of jet path on the morphology and mat size of synthetic polystyrene (PS) fibers during the electrospinning process. In addition, the mechanism of the fiber mats, which were prepared by varying the solution concentration, was evaluated. The straight jet length, envelope cone and whipping frequency of each electrospun jet were studied using images captured by a high-speed photography camera. The results showed that higher solution concentrations led to longer straight jet lengths, smaller envelope cones and lower whipping frequencies. The diameter and surface morphology of the PS fibers were also characterized by scanning electron microscopy (SEM). It was found that fibers spun with higher solution concentrations exhibited larger diameters and diameter distributions because of their jet path features. Furthermore, the electrospun jets with higher concentrations increased elongation and produced smaller fiber mats and higher breaking forces as a result of their different jet paths, which was a consequence of varying the solution concentration.

## 1. Introduction

Electrospinning, an efficient and popular process and versatile technique, can be used to fabricate nano- and sub-micron fibers from a wide array of polymers [1,2,3]. With the assistance of a high applied voltage and a grounded target, this process is capable of creating ultrafine polymer fibers. During the electrospinning process, the electric force generated by the electric potential charges a polymer solution, causing the polymer to undergo a shape change from a droplet to a cone, known as a “Taylor cone” [4,5]. The electrified jet, which has a diameter much smaller than the nozzle, is issued from the Taylor cone. The jet then bends into a whipping path after it flows away from the spinneret in a nearly-straight line, and it is during this process that the jet is stretched and thinned by the electrical force at very large ratios. The solvent then evaporates over the jet path, and thus polymer nanofibers are formed on the collector [6,7,8]. Both the large surface-to-volume ratio property of the electrospun fibers and the interconnected pores in the nanofibrous nonwovens enable the electrospun products to have a myriad of applications. These applications range from scaffolds for tissue engineering [9] to components of biosensors [10] and energy harvesting devices [11], and dental applications [12,13].

For better control over the morphology and properties of nanofibers, it is a matter of great significance to understand the role of the jet path in the electrospinning process [3,8,14,15]. Extensive research has been devoted to experimental evaluation of the effects of the solution, processing, and ambient parameters on the morphology of electrospun fibers [16,17,18]. Only a few of these studies have indicated that these parameters have a notable influence on the path of the electrospun jet, which further affects the morphology and properties of the resultant fibers. Yang et al. [19] designed three types of electrospinning setups to study the effects of the electric field distribution uniformity on the jet path. The experimental observations reported that the electric field distribution uniformity had a substantial effect on the jet motion and fiber diameter. Zeng et al. [17,20] analyzed the effects of the electric field on jet motion in a multi-jet electrospinning process, finding that both electric field strength and uniformity affected the jet path, thereby causing a deviation in the multi-jet electrospinning process. In addition, their results showed that both the strength and uniformity would influence the diameter and uniformity of the resultant fiber. Many studies [17,19] on jet paths focus on the electric field. Results of previous research on the solution parameters, such as concentration, molecular and solvent properties, show noteworthy influence of the solution on the fiber diameter, morphology and inner structure of the electrospun fibers [21,22]. In order to thoroughly understand the effects of the solution parameters on an electrospun fiber’s morphology, diameter and properties, systematic research on the jet path would need be taken into consideration.

In general, the jet path can be divided into two parts: Straight jet and whipping jet, both of which affect the formation of electrospun fibers. Variables such as the processing parameters, solution properties and the ambient conditions influence the fiber diameter and morphology, mainly because varying these parameters can induce different jet paths. At this time, only a few parameters, including the electric field strength and uniformity, have attracted the attention of researchers [15,18,20], despite the concentration of the polymer solution being a key factor in fiber diameter and mechanical properties, as well as the solution viscosity and conductivity [23]. Currently, although the important role of solution concentration has been recognized in experimental electrospinning studies, the effects of solution concentration on the jet path and resultant fiber performance have not been systematically studied [16,24].

The aim of this work was to explore the effects of the solution concentration on the jet path, resultant fiber morphology, fiber mat size and fiber mechanical properties. Polystyrene (PS) was adopted for this study because it is known to be prone to forming fibers with various morphologies [25,26] and has widespread use in filtration [27] and separation [28]. PS fibers were electrospun by varying the concentration of the PS polymer, and the jet paths of the polymer solutions with various concentrations were recorded by a high-speed camera. For the purpose of investigating the influences of jet path on fiber morphology, mat size and properties, the captured images were further analyzed by image processing techniques. Three parameters, including the straight jet length, envelope cone and whipping frequency of the jet, were measured to characterize the jet motion. In addition, the mechanical performance of the PS fiber mats was evaluated, in order to assess the effects of the jet path on the resultant fiber mats.

## 2. Experimental Section

### 2.1. Materials Preparation

The polystyrene (PS, (C_8_H_8_)*_n_*, weight-average molecular weight, *M*w ~ 350,000 g/mol, SIGMA-ALDRICH Co., Saint Louis, MO, USA) and *N,N*-Dimethylformamide (DMF, analytical grade, C_3_H_7_NO, molecular weight of 73.09, Sinopharm Chemical Reagent Co., Ltd., Shanghai, China) were used as received. The PS solutions at different concentrations (10, 15, 20, 25 and 30 wt %) were prepared by dissolving PS powders into DMF solutions, followed by gently stirring for 5 h with an electric mixer (JB90-D, Shanghai Specimen and Model Factory, Shanghai, China) at specified temperature and humidity (25 ± 0.2 °C, 40% ± 5%).

### 2.2. Experimental Setup

**Figure 1:**
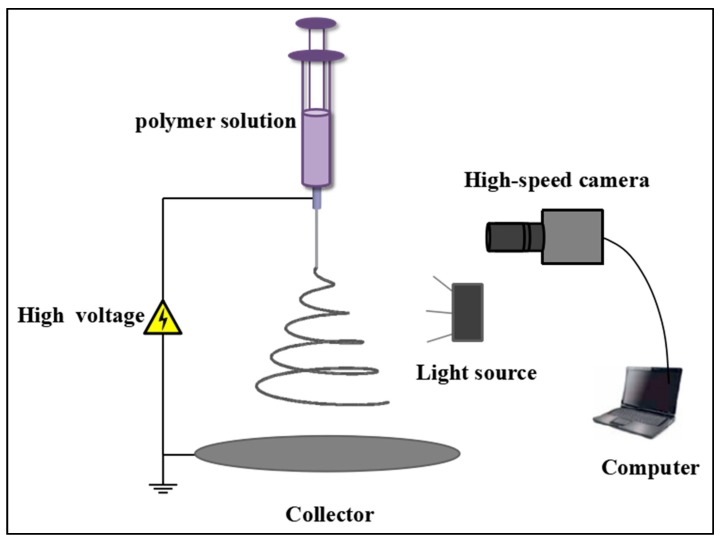
Schematic of experimental setup.

The setup of the single metal needle for the electrospinning process is shown in Figure 1. The metal needle (Shenzhen Baohao Metal Materials Co., Ltd., Shenzhen, China), with an outer/inner diameter of 0.9 mm/0.6 mm and length of 13 mm, was a blunt-tip stainless-steel needle, powered by a high voltage. The polymer solution was forced from a syringe driven by a pump (LD-P2020, Shanghai Lande Ltd., Shanghai, China) into the needle, with flow rate of 1.0 mL/h. A high voltage power supply (ES-60P 20 W, Gamma High Voltage Research Inc., San Francisco, FL, USA) was applied to the spinneret and aluminum foil-grounded collector, with the distance between them being set at 20 cm.

The jet motion during the electrospinning process was captured by means of high-speed photography. A Photron Fastcam Mini AX200 high-speed camera (Photron Ltd., Tokyo, Japan) equipped with a Tokina 100 mm f 2.8 macro lens, capable of recording images at a frame rate up to 900,000 frames per second (fps), was employed to capture jet motion. Two 100 W lamps (Model-100LED TWIN, Shanghai Jinqiaojingyi High-tech Co., Ltd., Shanghai, China) were used as the light source. Image processing and analysis, managed by Adobe Photoshop, Image J and the software supplied with the camera, was conducted to analyze the jet paths.

All experiments were carried out in a controlled environment, with a temperature of 25 °C and a relative humidity of 50% ± 5%.

### 2.3. Characterization

The viscosities and conductivities of the polymer solutions were measured by a viscometer (NDJ-79, LICHEN, Shanghai, China) and a conductometer (ExStik, EXTECH, Waltham, MA, USA), respectively, with each of these two parameters being measured three times. In addition, a micrometer (Art.Nr.64200, MASTERPROOF, Berlin, Germany) was used to obtain the thickness of the nanocomposite mats by performing measurements every centimeter along the center line of the fiber mats. The PS fibers were then coated with gold, allowing observation of their surface morphology by a SEM (Field Emission Scanning Electron Microscope, SU8010, Hitachi, Japan) at an accelerating voltage of 5 kV. The diameters of the fibers were calculated by image visualization software (ImageJ, NIH Image, Bethesda, MD, USA). Finally, the mechanical properties of the electrospun fiber mat samples (50 mm × 10 mm) were tested by a fiber strength tester (XS (08) XT-2, Shanghai, China) at the speed of 10 mm/min, with each sample being measured 5 times.

## 3. Results and Discussion

### 3.1. PS Solution Performance

**Figure 2:**
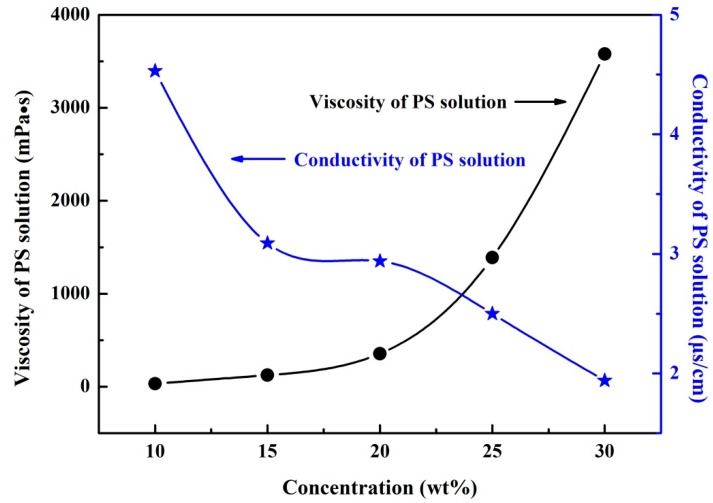
Viscosity and conductivity of polystyrene (PS) solutions as a function of concentration.

When the solution concentration directly affects the entanglement degree of the polymer molecular chain in the solution and plays a determinative role in the solution viscosity, it is considered to be one of the most essential parameters required for the electrospinning process. In addition, the polymer solution must be of a certain electrical conductivity so that it is capable of polarizing the solution at the spinneret tip to form a jet. Figure 2 shows the viscosities and conductivities of the PS solutions with different concentrations. As the concentration of the PS solution increases, the viscosity also increases; this is because solutions with higher polymer concentrations obtain greater entanglement of polymer molecular chains. As can be seen in Figure 2, the conductivity of a PS solution is mainly dependent on the solvent content, which decreases with increasing polymer concentration; i.e., as the polymer content in the solution increases, the conductivity decreases.

### 3.2. Jet Path Observations and Measurements

Typically, the electrospun jet has an almost straight line followed by a number of bending loops. Whereas the straight section can be observed under a light source using the naked eye, it is impossible to catch the bending part of the jet due to the high speed of whipping [8,17]. In this study, in order to get better observation of the bending jet, a high-resolution, high-speed camera was employed to capture the trajectory of the electrospinning jet.

Experiments were performed under the same conditions, with 20 kV electrospinning voltages, 20 cm working distance, 25 °C ambient temperature and 50% ± 5% humidity. However, the solution concentration was changed from 10 to 30 wt %. The straight section and whipping jet were recorded at 6400 fps with 1024 pixels × 1024 pixels, showing the time evolution of the bending jets in detail.

**Figure 3:**
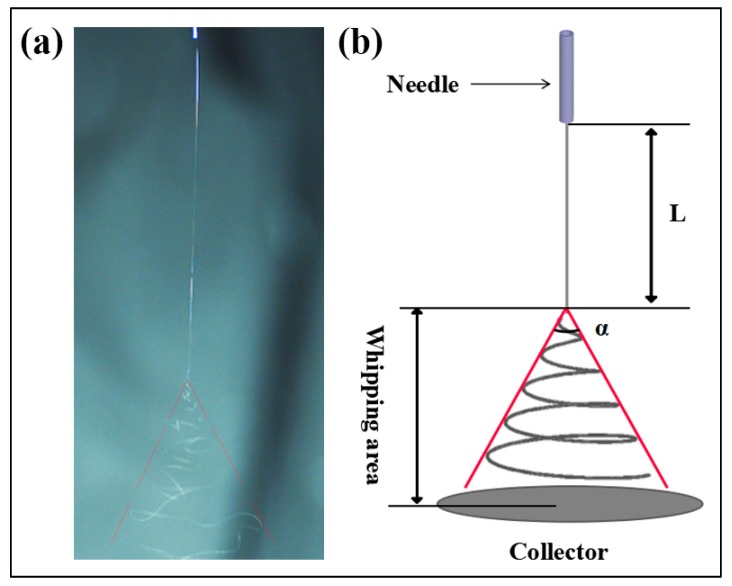
(**a**) A high-speed photograph of jet motion at 6400 fps and (**b**) jet path schematics of the electrospinning process.

Three parameters, including the straight jet length, envelope cone and whipping frequency—established based on the images captured by the high-speed camera—were used for the characterization of jet motion. A typical image of the jet motion taken by the high-speed camera is shown in Figure 3a. The straight jet length, L, and the envelope cone, α, may be defined as shown in Figure 3b. Specifically, the straight jet length is defined as the straight section of the jet path. The envelope cone was measured to compare the extent of jet whipping with different solution concentrations. The whipping frequency, ω, is defined as the rate which new bends are generated at the onset of instability, with units rev/s. The number of bending loops, BN (revolution), was counted over a certain period, *t*, using the high-speed camera images at 6400 fps, which then allowed calculation of ω using [19]:

(1) $$\omega =\frac{\mathrm{BN}}{t}$$

The mean ω values were calculated on the basis of 800 images of random samples captured by the high-speed camera.

**Figure 4:**
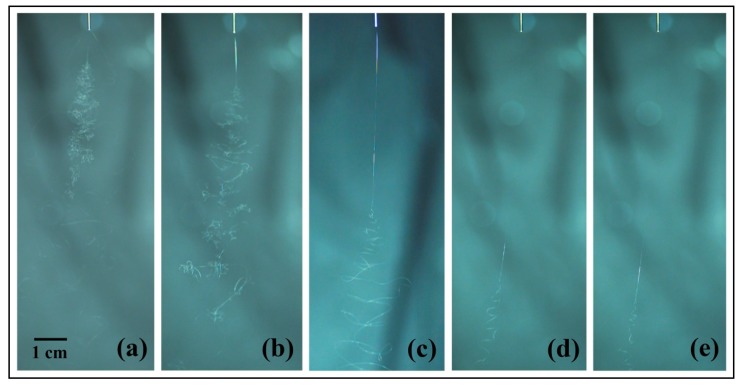
Jet trajectories captured by high-speed photography at different solution concentrations: (**a**) 10 wt %; (**b**) 15 wt %; (**c**) 20 wt %; (**d**) 25 wt %; and (**e**) 30 wt %. The electrospinning processing parameters were as follows: Applied voltage of 20 kV, working distance of 20 cm, and flow rate of 1.0 mL/h.

**Figure 5:**
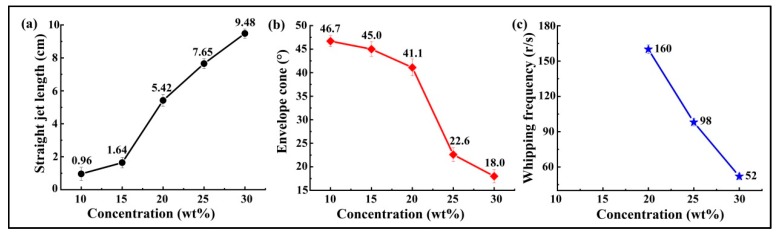
Whipping parameters at different solution concentrations: (**a**) Straight jet length; (**b**) envelope cone; and (**c)** whipping frequency. The electrospinning processing parameters were as follows: Applied voltage of 20 kV, working distance of 20 cm, and flow rate of 1.0 mL/h.

Figure 4 shows the jet motions of the PS solutions at various concentrations. It may be observed that a series of bending loops were generated after the straight section, which increased in length as the solution concentration was increased. During experimentation, it was difficult to get a clear picture of the jet trajectory with 10 and 15 wt % PS solutions due to the bending instability. Furthermore, it was found that the jet splits at the needle tip for the solution concentration 10 wt %. Figure 5 depicts the results obtained for straight jet length, envelope cone and whipping frequency of the jets, for the various solution concentrations. As the concentration was increased from 10 to 30 wt %, the straight jet length increased from 0.96 to 9.48 cm (Figure 5a), while the envelope cone decreased from 46.7° to 18° (Figure 5b). For the solutions at concentrations of 10 and 15 wt %, the whipping frequencies of the jets failed to be calculated due to the jet’s bending instability; no completed loop could be identified from the images. The main reason for this instability was the solutions with lower concentrations possessed lower viscosities and higher electrical conductivities (as shown in Figure 2), which means the jets were carrying more charges. Thus, the jets with low solution concentration were subject to strong electric field forces and surface charge repulsion in the spinning process, causing more jet whipping, larger jet whipping angles and shorter straight-line segments. As the solution concentration was increased, the resulting electric field force and the surface charge repulsion force of the jet was decreased. As a result, the jets with the higher solution concentrations were less prone to bend, which resulted in longer straight jets and smaller envelope cones.

### 3.3. Fiber Diameters and Fiber Mats

**Figure 6:**
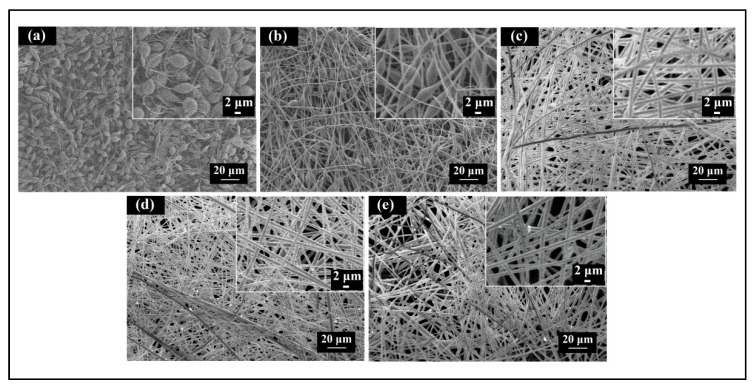
Scanning electron microscopy (SEM) images of electrospun PS fibers formed at different solution concentrations: (**a**) 10 wt %; (**b**) 15 wt %; (**c**) 20 wt %; (**d**) 25 wt %; and (**e**) 30 wt %. The processing parameters were as follows: Applied voltage of 20 kV, working distance of 20 cm, and flow rate of 1.0 mL/h.

The scanning electron microscopy (SEM) images of the electrospun PS fibers fabricated at various solution concentrations are shown in Figure 6. The fibers formed from the PS solutions at concentrations of 10 and 15 wt % exhibited beads-on-a-string morphology (Figure 6a,b). As the concentration of the solution was increased, the fibers became smoother and lost their beading components. Previous studies have indicated that various structures of electrospun PS fibers, including beads, grooves and pores, can be obtained by changing the polymer concentrations to induce the unstable fluid jet formation [29,30,31]. As shown in Figure 4a,b, the unstable trajectories of the electrospun jets formed from PS solutions at concentrations of 10 and 15 wt % may be the main reason for the beads-on-a-string fiber morphology, which may be further supported by the results depicted in Figure 5a,b. The jets with 10 and 15 wt % concentrations had average straight jet lengths of 0.96 and 1.64 cm, and whips with 46.7° and 45.0° envelope cones, respectively. The straight jet part was a stable region for jet stretching, however, the straight jet lengths corresponding to these two concentrations were much lower than those of others. Furthermore, the larger whipping envelope cone indicated more intense jet instability. The lower concentration solution jet also carried more charges and was subject to stronger electric field forces and surface charge repulsion, which could have led to jet instability and formation of beads due to insufficient stretch.

**Figure 7:**
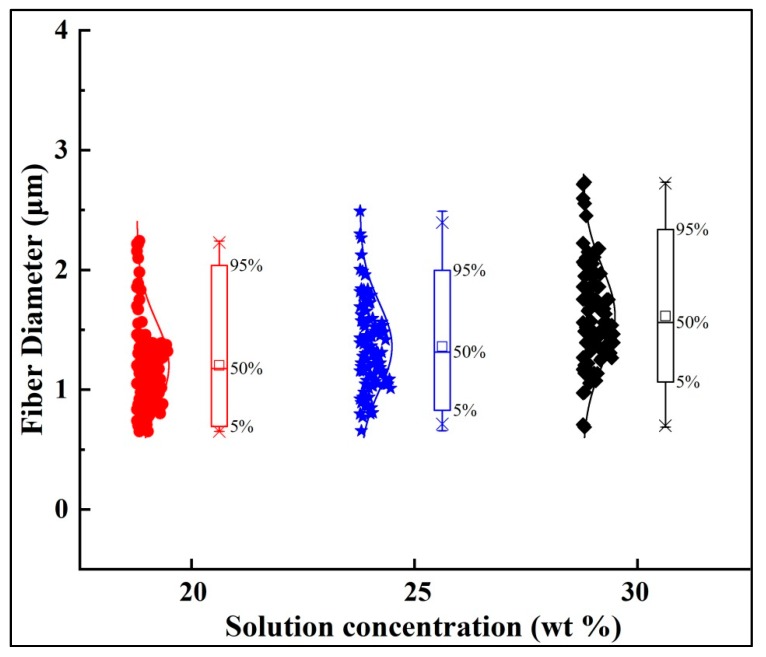
Diameters of fibers prepared by solutions at diverse concentrations. The processing parameters are as follows: Applied voltage of 20 kV, working distance of 20 cm, and flow rate of 1.0 mL/h.

Figure 7 shows the diameter distributions of the fibers electrospun with 20, 25, and 30 wt % solution concentrations. Each point in Figure 7 indicates one individual measurement of a single fiber section obtained from a single sample. The box and bar on the right of the figure illustrates the distribution of the fiber diameter, and the small box represents the average diameter. It can be observed that under the same processing conditions, lower solution concentration produced finer fibers with smaller diameter distributions. It is well known that jet bending is the main factor responsible for the reduction of fiber diameter during the electrospinning process. Different solution concentrations result in different fiber diameters and fiber morphology, due to different jet paths. The results displayed in Figure 4 and Figure 5 show that the jet path with a higher solution concentration has a longer straight jet, smaller bending area (smaller envelope cone, shorter bending height), and lower whipping frequency. Consequently, larger bending area and speed helped to produce finer fibers due to additional stretching. Furthermore, in the case of lower solution concentration, less polymer contents could be drawn from the syringe. All these factors contributed to the finer fibers produced by the lower polymer solution.

**Figure 8:**
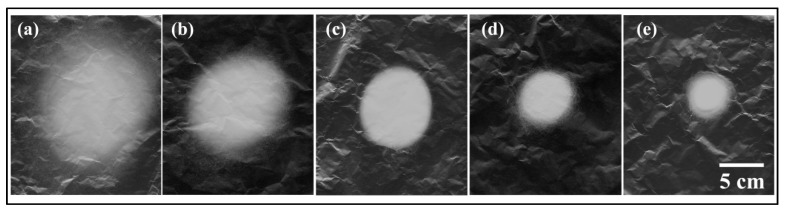
Fiber mats collected from five different solution concentrations by flat collectors: (**a**) 10 wt %; (**b**) 15 wt %; (**c**) 20 wt %; (**d**) 25 wt %; (**e**) 30 wt %. The collecting time is set to be 10 min. The processing parameters are as follows: Applied voltage of 20 kV, working distance of 20 cm, and flow rate of 1.0 mL/h.

**Figure 9:**
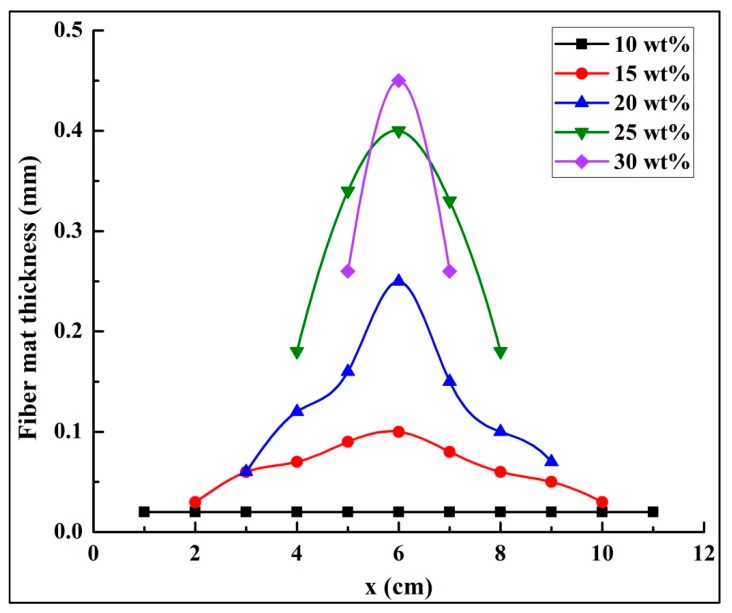
Fiber mat thicknesses at central line, collected for five different solution concentrations. The collecting time was set at 120 min. The processing parameters were as follows: Applied voltage of 20 kV, working distance of 20 cm, and flow rate of 1.0 mL/h.

Figure 8 demonstrates the deposited patterns of fiber mats collected from different solution concentrations after a 10 min spinning period. Evidently, the higher solution concentrations resulted in smaller fiber mats; this was because the jets with lower solution concentrations created longer straight jets, smaller envelope cones and lower whipping frequencies in the same spinning distance. Figure 9 shows the thicknesses of the fiber mats electrospun at different PS concentrations after 120 min. The thickness of the mat was measured every centimeter along the central line of the fiber mat (i.e., the *x*-direction). As the solubility of the solution was increased, the thickness of the fiber mats increased. From these results, it is evident that it is easier to produce a mat with uniform thickness using lower polymer concentrations.

### 3.4. Mechanical Performance of PS Fiber Mats

**Figure 10:**
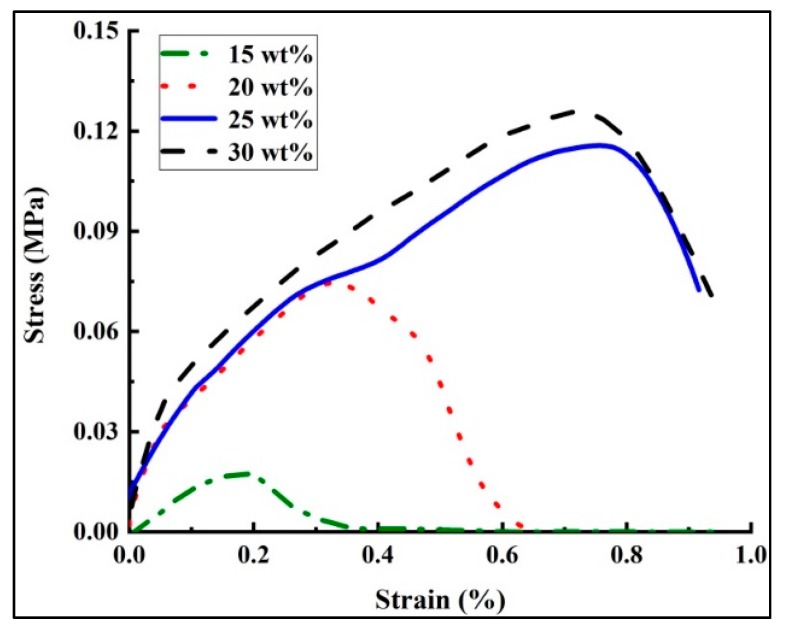
Stress–strain curves of different solution concentrations. The collecting time of the fiber mats was set to 120 min. The processing parameters were as follows: Applied voltage of 20 kV, working distance of 20 cm, and flow rate of 1.0 mL/h.

After generating the PS fiber mats via a dynamic drum collector for obtaining uniform fiber mats, the tensile properties of the resulting mats—created at different solution concentrations—were investigated for this study [32]. The five fiber mats, with size 50 mm × 10 mm, were tested by the fiber strength tester for each solution concentration. The spinning time for these samples was set to be 120 min. The typical stress–strain curves for each fiber mat are shown in Figure 10. It may be observed that the stress increased as the strain increased, and the fiber mats produced by higher solution concentrations attained higher maximum stress or peak stress. The stress–strain curves showed an increase of maximum stress from about 0.015 to 0.12 MPa with increasing solution concentration. As mentioned above, an increase in the solution concentration could lead to an increase in the polymer molecular contents, which could, in turn, generate stronger fibers. The maximum strain increased from about 0.4% to 0.95% with an increase in the solution concentration (15 to 30 wt %). The jets with lower solution concentrations had a shorter straight jet and larger bending area, which could have provided more sufficient chain stretching and thus resulted in smaller breaking elongation. The maximum stress and strain of the solution at the concentration of 15 wt % were much smaller than that of other concentrations, mainly because of the beads on string morphology. The slope of the stress–strain curves characterize the modulus of the fiber mats. The modulus (stiffness) of mats was heavily dependent on the solution concentration, as it increased with the concentration. The beads-free fibers with larger fiber diameters were obtained from the solutions with higher concentrations, which lead to stiffer fiber mats. The modulus depending on the fiber diameter agrees with previously published data [33]. As for the 10 wt % solution concentration, the fiber mats prepared at the same spinning time (120 min) was too thin to be taken off of the collecting foil.

## 4. Conclusions

In this work, a comprehensively-designed study was correctly performed to explore the effects of jet path on the performance of both electrospun fibers and fiber mats. The observation results from the images captured by a high-speed camera show that the jet path was influenced by the solution concentration. Experimental results have indicated that a higher solution concentration results in a longer straight jet, smaller envelope cone and lower bending frequency, which brings about smaller fiber diameters and smaller fiber mats. The images of the jet paths at lower concentrations (10 and 15 wt %) explains the reason for the beads-on-a-string morphology of PS fibers. In addition, the study showed that the electrospun PS fiber mats with higher solution concentrations obtained better mechanical properties, which could provide the basis for research applications. The effects of the solution concentration on the internal structure of the electrospun jets and the resultant fibers are not discussed in this paper, and need further investigation.
